# Paying for Telemedicine in Smaller Rural Hospitals: Extending the Technology to Those Who Benefit Most

**DOI:** 10.1001/jamahealthforum.2021.1570

**Published:** 2021-08-03

**Authors:** Kori S. Zachrison, Jessica V. Richard, Ateev Mehrotra

**Affiliations:** Department of Emergency Medicine, Massachusetts General Hospital, Boston; Department of Emergency Medicine, Harvard Medical School, Boston, Massachusetts; Department of Health Care Policy, Harvard Medical School, Boston, Massachusetts; Department of Health Care Policy, Harvard Medical School, Boston, Massachusetts; Department of Medicine, Beth Israel Deaconess Medical Center, Boston, Massachusetts; Department of Medicine, Harvard Medical School, Boston, Massachusetts

Emergency departments (EDs) and hospitals in the US vary dramatically in their access to specialty input. Patients presenting to smaller EDs are increasingly transferred to urban referral centers for specialty care.^1,2^ Telemedicine provides an opportunity to counter this trend. By bringing a consulting specialist virtually to the patient’s bedside, telemedicine may enable patients to receive the care they need closer to home. In doing so, smaller hospitals struggling to stay open may be able to retain more patients and improve their financial footing.

Approximately half of US hospital EDs now use telemedicine.^3^ The paradox is that smaller rural EDs are the most likely to benefit from telemedicine, but the least likely to have it.^3^ Telestroke care (ie, telemedicine for acute stroke care) provides a useful case study; it is associated with improved care quality and outcomes, with the greatest benefit to patients in lower-volume hospitals.^4^ Yet, smaller rural critical access hospitals are less likely to use telestroke care compared with larger, suburban community hospitals.^5^ This creates substantial equity issues and may contribute to the higher stroke morbidity and mortality seen among rural patients. In this Viewpoint, we describe the barriers to introducing telemedicine at smaller rural hospitals and explore potential policy solutions.

## Telemedicine Financing

Many hospitals receive telemedicine services as part of an academic center’s network, while other hospitals contract with private companies. Hospital EDs may use telemedicine for a single condition (eg, stroke) or for a range of conditions, and hospitals are increasingly using it for inpatients as well.

Hospitals that implement telemedicine must pay the initial fixed costs for equipment and internet connectivity, which according to a study published in 2015 can range from $17 000 to $50 000.^6^ Once set-up is complete, hospitals may contract on a per consultation basis or per condition (eg, mental health) with unlimited consultations. The same 2015 study found that the average annual subscription fee was $60 000, and that connectivity and maintenance expenses were an additional $3000 to $8000 per year.^6^

Typically, ED visits are reimbursed via a facility payment to the ED where the patient was seen and a professional payment to the on-site clinician. There is no additional payment to the ED for a telemedicine consultation. Rather, payers (eg, Medicare) reimburse the remote specialist-consultant. The academic hospital or private company providing the telemedicine can bill for each consultation to reduce the cost of the subscription service; however, surprisingly few ED telemedicine consultations are submitted for reimbursement, and most EDs simply pay for the subscription costs as overhead.

In addition to direct reimbursement, there are other benefits for a hospital that offset upfront telemedicine and subscription costs. Telemedicine may reduce both the need for physician recruitment^6^ and the risk of malpractice. Returning to the example of telestroke care, telestroke availability may be associated with a decreased risk of a malpractice suit for use of thrombolytic therapy. Telemedicine may also raise hospital revenues by deferring transfers of patients, and thereby increasing admissions. Hospitals that avoid transferring patients covered by Medicare receive the full diagnosis-related group payment rather than a per-diem fraction. The potential benefits of deferred transfers are less clear for critical access hospitals because they are paid on a cost basis and have restrictions on the length of hospital admissions.

## Barriers to Telemedicine Use

There are a number of barriers to using telemedicine at small rural hospitals. First, whereas larger hospitals can accept telemedicine expenses as part of the cost of doing business, smaller rural hospitals often lack the adequate cash flow and may not see its financial benefit given their low volumes of patients. Whether telemedicine is associated with a reduction in the number of transfers at smaller hospitals is less clear because a transfer may still be required after a telemedicine consultation (ie, for additional diagnostic testing and treatment).

A second barrier to telemedicine at small rural hospitals is the low number of patients, which makes it difficult to obtain a return on investment because the fixed costs are distributed among fewer individuals. Many small remote hospitals treat very few patients with stroke in a year, and reimbursement for a handful of consultations does not cover the full operating expenses of telemedicine. A low volume of patients is also an issue for the consulting telemedicine organization, either an academic hub or a private company, because it is harder to recuperate the upfront investment cost (eg, contractual expenses, credentialing, maintenance).

A third barrier, also related to the low volume of patients, is that repetitive use is required to become adept at using a new technology. Although telemedicine equipment (eg, the camera, software) is becoming more user friendly, if only 1 (or fewer) telemedicine consultation is necessary per month, clinicians may be less inclined to use the technology.

Fourth, it is difficult to recuperate expenses using the current reimbursement systems. As noted, EDs pay for the telemedicine service, but the reimbursement goes to the telemedicine consulting specialist. While EDs can bill on behalf of clinicians, which would defray subscription costs, few do so because the administrative barriers of credentialing and licensure are prohibitive across multiple insurers, especially given the low volume of consultations and the amount of reimbursement per visit.

## How to Move Forward

A number of policy interventions could help. First, for acute care services, reimbursement could be made directly to the ED where the patient was seen. This payment model is more consistent with how hospitals pay for programs and would enable a more straightforward cost-benefit analysis for sites that are considering deploying telemedicine.

Second, payment for telemedicine consultations at smaller hospitals could be increased. Given that many costs are fixed, the average cost per patient of a given telemedicine consultation at a small rural hospital is much higher than at a higher-volume center. Expanding on existing programs,^7^ smaller hospitals could also receive subsidies to defray costs of infrastructure and contracting.

Third, coordinating centers for smaller hospitals could pool demand and consolidate efforts. This strategy could mimic that of vaccine-purchasing groups, which contract directly with manufacturers on behalf of many medical practices. The upfront costs of the technology would remain for each site; however, contracting services as a collaborative may generate a cumulative volume and demand that would be a more worthwhile venture for the telemedicine-providing organization. Coordinating centers could also work on behalf of many hospitals to centralize credentialing and help with service contracting.

This coordination model could be built into the 12 regional resource centers supported by the Health Resources and Services Administration.^8^ These centers provide resources for telehealth education, training, and implementation assistance, including tools for navigating regulations and reimbursement policy. These centers could be expanded to consolidate administrative load and aggregate the number of patients from smaller rural sites in their region.

Academic hospitals could also play this coordinating role. Many telemedicine programs are already run by academic centers. For example, the University of California Davis has more than 200 sites^9^ and the University of Utah supports more than 80 sites.^10^ These programs are often consistent with the missions of the academic institutions, and expansion of grants and funding to the smallest rural sites could address their current gaps.

Smaller rural hospitals where patients could benefit the most from telemedicine are the least likely to use this technology. In large part this is owing to the cost of telemedicine and the limitations of current payment models. The policy solutions proposed in this Viewpoint may help to ensure that patients who seek care at small rural hospitals are not left behind by the shift to virtual care.
